# Supplementary material for: The Application of Arsenic Trioxide in Ameliorating ABT-737 Target Therapy on Uterine Cervical Cancer Cells through Unique Pathways in Cell Death
Source: Cancers (Basel). 2019 Dec 31;12(1):108. doi: 10.3390/cancers12010108 (PMC7016694; doi:10.3390/cancers12010108)
Supplement: Supplementary file 1 [file cancers-12-00108-s001.pdf]

Supplementary Materials

MEF

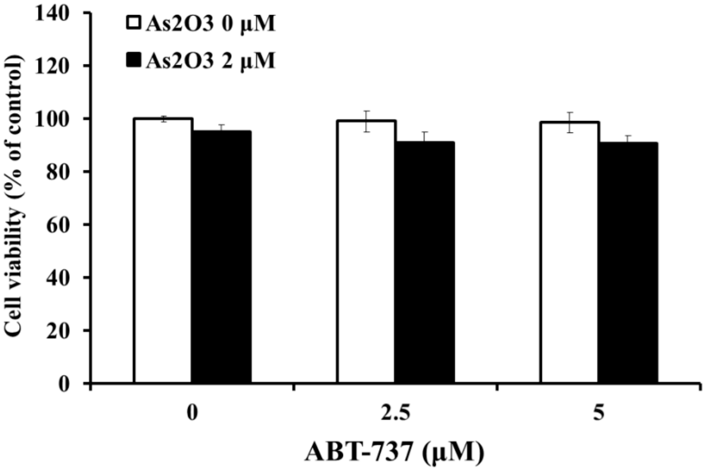

HaCaT

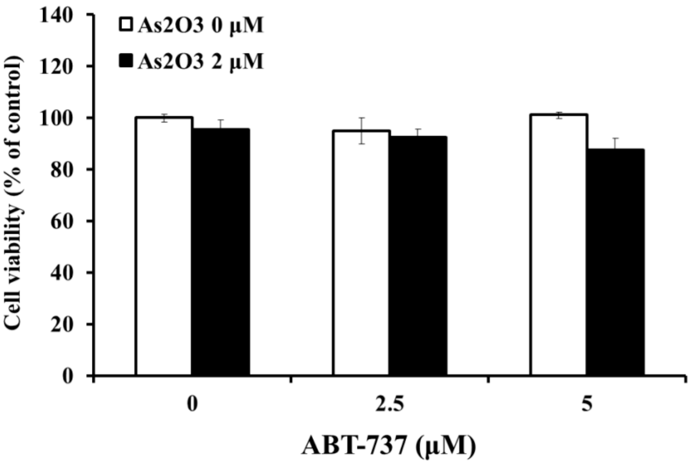

Figure S1. Effect of ABT-737 and As_2O_3 on cell viability in Mouse Embryonic Fibroblasts (MEF) and human keratinocyte cell line, HaCaT. MEF and HaCaT (8×10^3 cells) were treated with ABT-737 and As_2O_3 for 48 h. The cell viability was analyzed using MTT assay.

SiHa

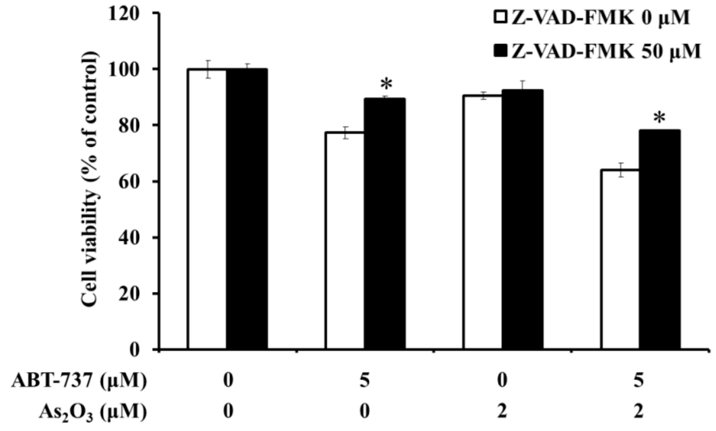

Caski

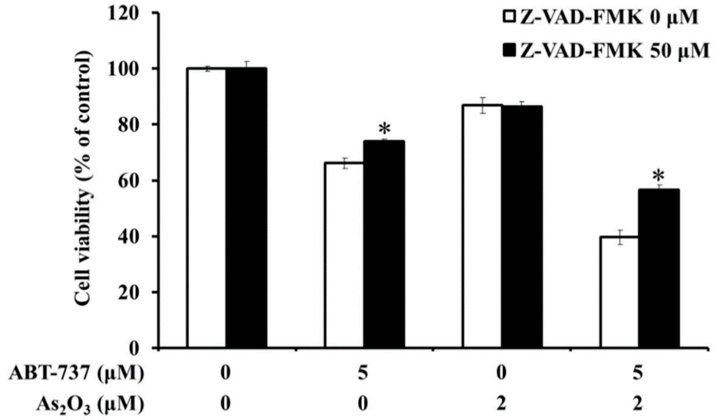

Figure S2. Effect of pan-caspase inhibitor Z-VAD-FMK on cell viability in SiHa and Caski cells after ABT-737 and As_2O_3 treatment**.** SiHa and Caski (5×10^3 cells) were treated with Z-VAD-FMK, ABT-737 and As_2O_3 for 48 h. The cell viability was analyzed using MTT assay. * $p < 0.05$.

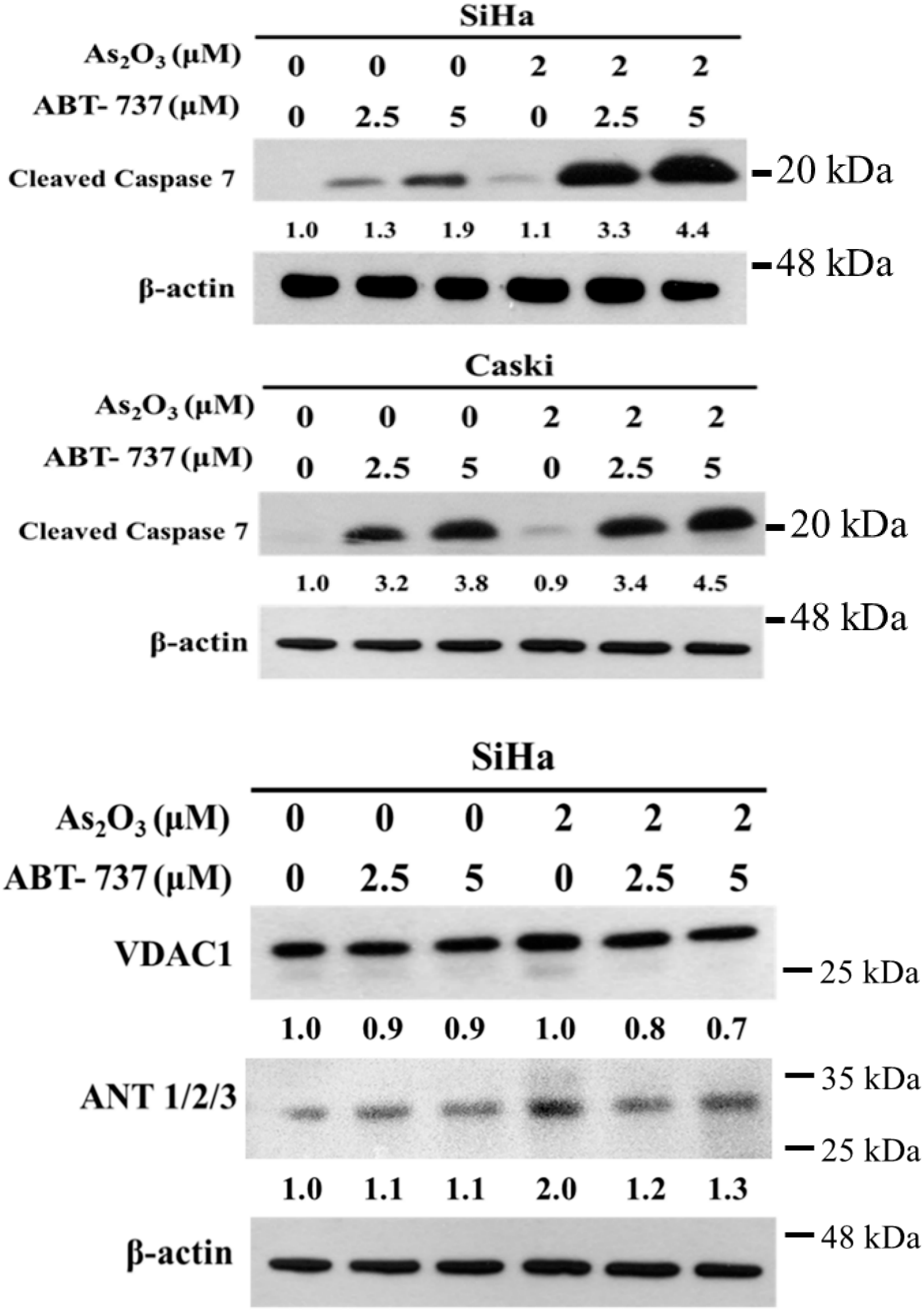
SiHa
As2O3 (μM) 0 0 0 2 2 2
ABT- 737 (μM) 0 2.5 5 0 2.5 5
Cleaved Caspase 7
20 kDa
1.0 1.3 1.9 1.1 3.3 4.4
β-actin
48 kDa
Caski
As2O3 (μM) 0 0 0 2 2 2
ABT- 737 (μM) 0 2.5 5 0 2.5 5
Cleaved Caspase 7
20 kDa
1.0 3.2 3.8 0.9 3.4 4.5
β-actin
48 kDa
SiHa
As2O3 (μM) 0 0 0 2 2 2
ABT- 737 (μM) 0 2.5 5 0 2.5 5
VDAC1
25 kDa
1.0 0.9 0.9 1.0 0.8 0.7
ANT 1/2/3
35 kDa
25 kDa
1.0 1.1 1.1 2.0 1.2 1.3
β-actin
48 kDa

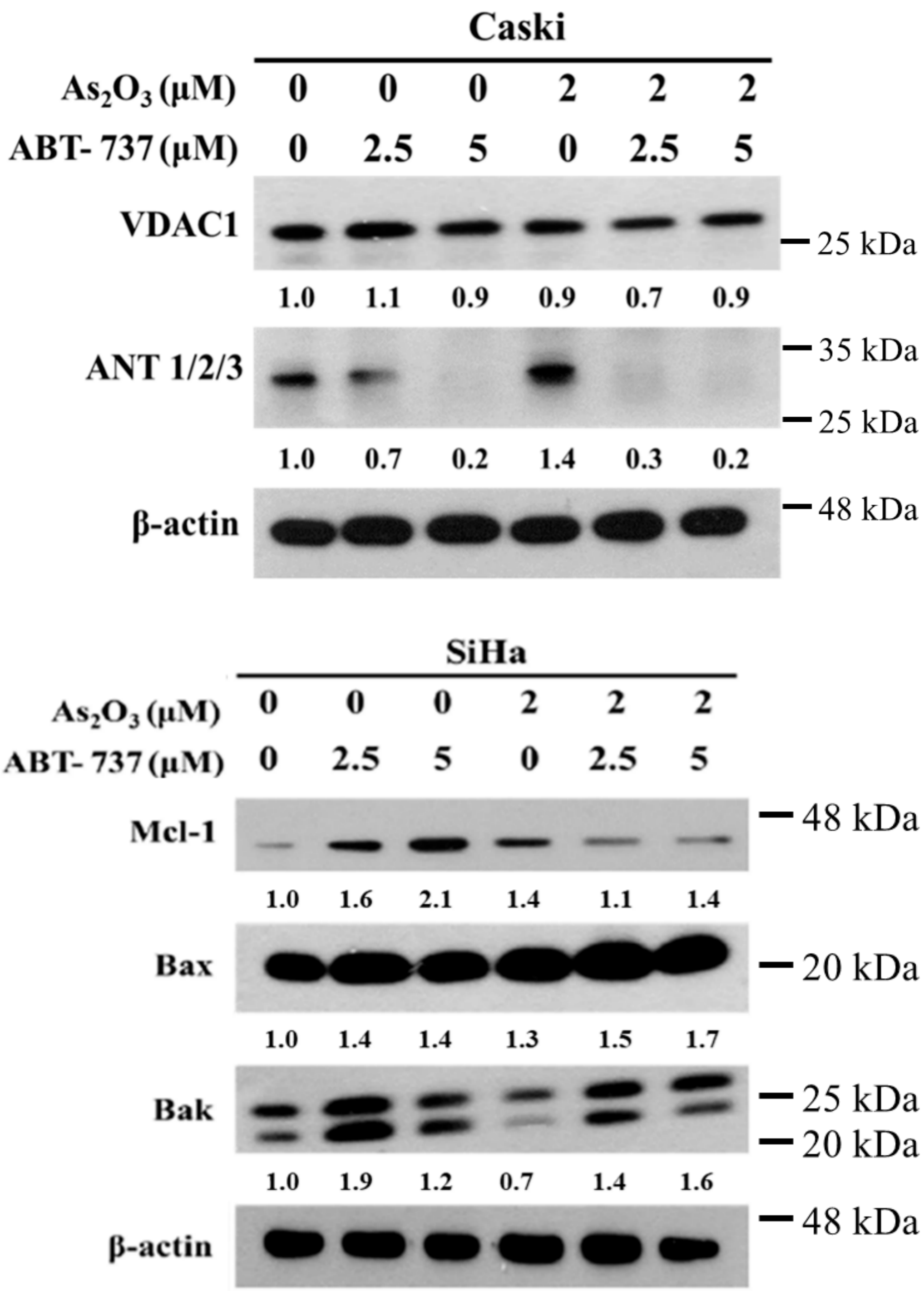
Caski
As2O3 (μM) 0 0 0 2 2 2
ABT- 737 (μM) 0 2.5 5 0 2.5 5
VDAC1
25 kDa
1.0 1.1 0.9 0.9 0.7 0.9
ANT 1/2/3
35 kDa
25 kDa
1.0 0.7 0.2 1.4 0.3 0.2
β-actin
48 kDa
SiHa
As2O3 (μM) 0 0 0 2 2 2
ABT- 737 (μM) 0 2.5 5 0 2.5 5
Mcl-1
48 kDa
1.0 1.6 2.1 1.4 1.1 1.4
Bax
20 kDa
1.0 1.4 1.4 1.3 1.5 1.7
Bak
25 kDa
20 kDa
1.0 1.9 1.2 0.7 1.4 1.6
β-actin
48 kDa

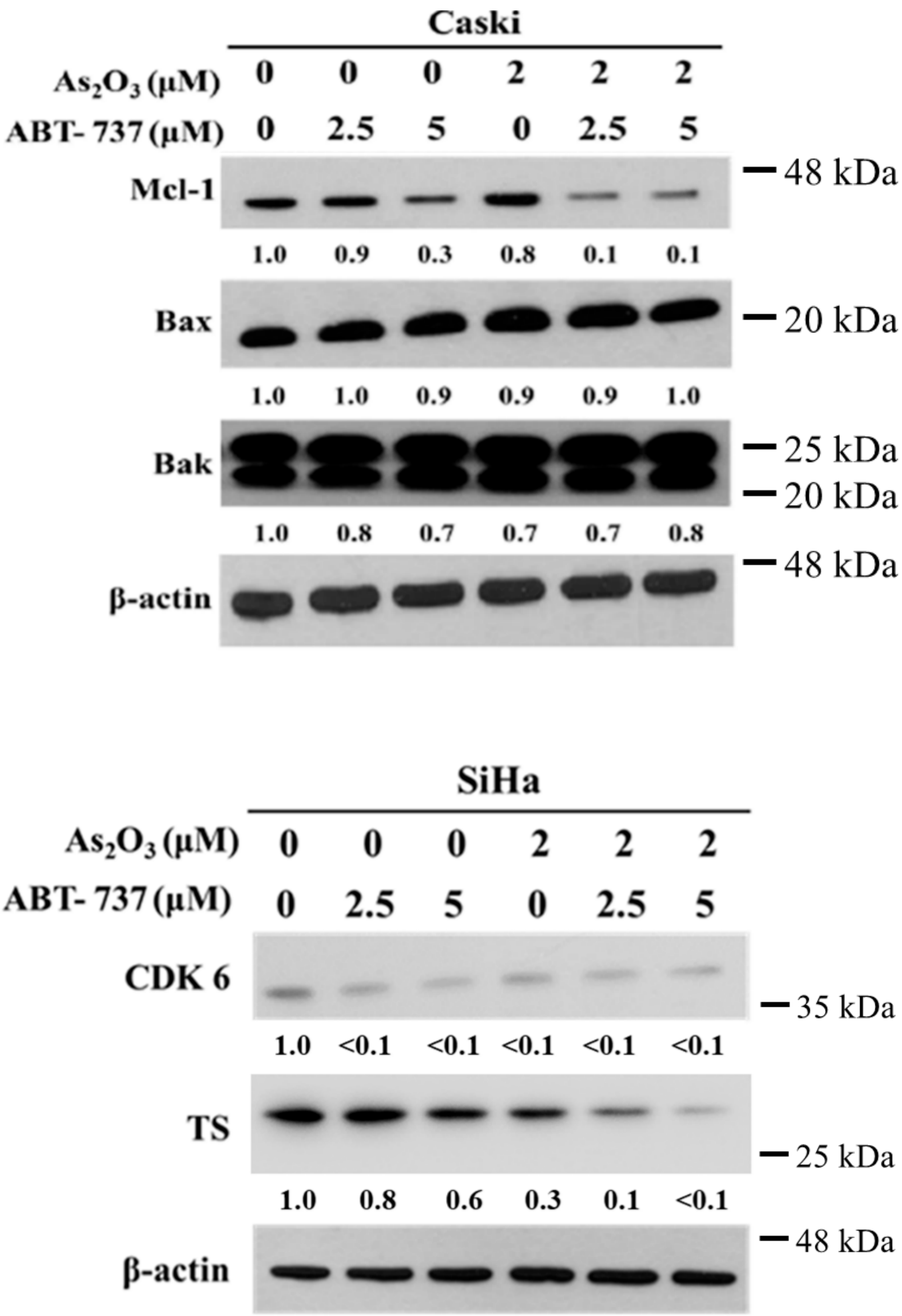

Caski
As2O3 (μM) 0 0 0 2 2 2
ABT- 737 (μM) 0 2.5 5 0 2.5 5
Mcl-1
48 kDa
1.0 0.9 0.3 0.8 0.1 0.1
Bax
20 kDa
1.0 1.0 0.9 0.9 0.9 1.0
Bak
25 kDa
20 kDa
1.0 0.8 0.7 0.7 0.7 0.8
β-actin
48 kDa
SiHa
As2O3 (μM) 0 0 0 2 2 2
ABT- 737 (μM) 0 2.5 5 0 2.5 5
CDK 6
35 kDa
1.0 <0.1 <0.1 <0.1 <0.1 <0.1
TS
25 kDa
1.0 0.8 0.6 0.3 0.1 <0.1
β-actin
48 kDa

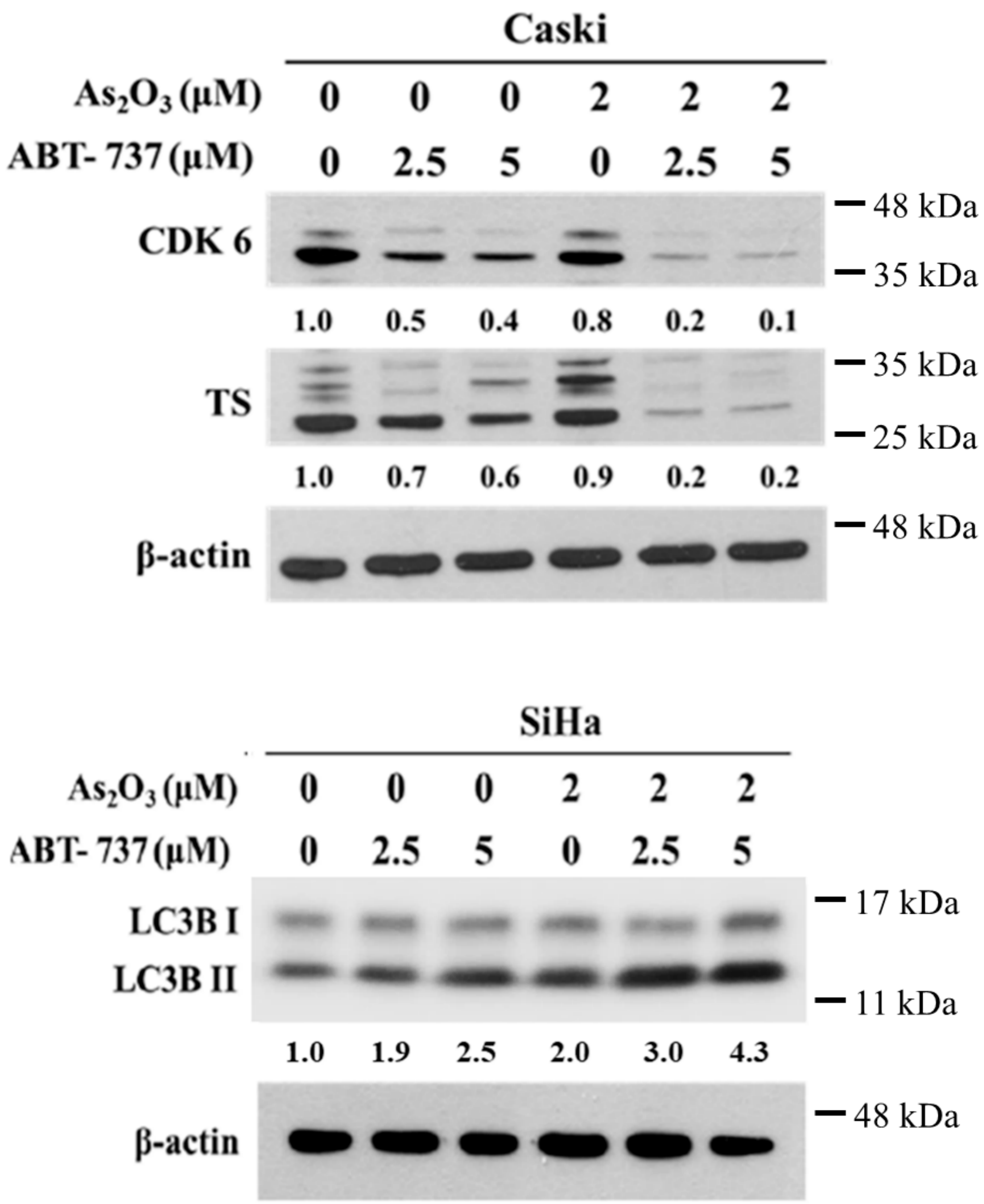
Caski
As2O3 (μM) 0 0 0 2 2 2
ABT- 737 (μM) 0 2.5 5 0 2.5 5
CDK 6
48 kDa
35 kDa
1.0 0.5 0.4 0.8 0.2 0.1
TS
35 kDa
25 kDa
1.0 0.7 0.6 0.9 0.2 0.2
β-actin
48 kDa
SiHa
As2O3 (μM) 0 0 0 2 2 2
ABT- 737 (μM) 0 2.5 5 0 2.5 5
LC3B I
LC3B II
17 kDa
11 kDa
1.0 1.9 2.5 2.0 3.0 4.3
β-actin
48 kDa

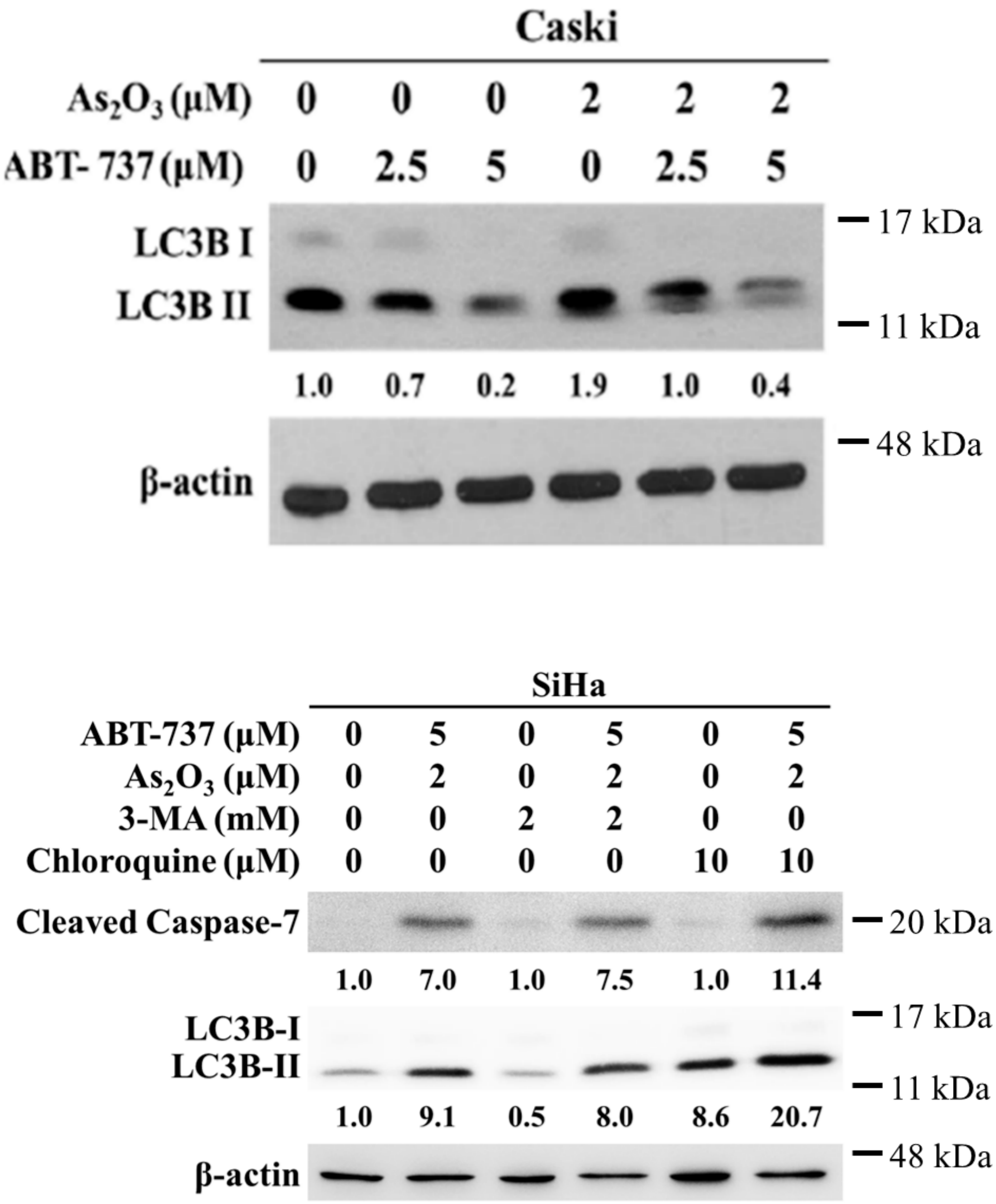

Figure S3. Wetern blot.
